# Differences between Health and Non-Health Science Students in Lifestyle Habits, Perceived Stress and Psychological Well-Being: A Cross-Sectional Study

**DOI:** 10.3390/nu16050620

**Published:** 2024-02-23

**Authors:** Mario Marendić, Diana Aranza, Ivan Aranza, Silvija Vladislavić, Ivana Kolčić

**Affiliations:** 1 University Department of Health Studies, University of Split, 21000 Split, Croatia; daranza@ozs.unist.hr (D.A.); svladislav@ozs.unist.hr (S.V.); 2 Institute of Emergency Medicine of Split-Dalmatia County, 21000 Split, Croatia; aranza.ivan@gmail.com; 3 School of Medicine, University of Split, 21000 Split, Croatia; ikolcic@mefst.hr; 4 Algebra LAB, Algebra University College, 10000 Zagreb, Croatia; 5 Psychiatric Clinic Sveti Ivan, 10000 Zagreb, Croatia

**Keywords:** students, lifestyle habits, perceived stress, wellbeing

## Abstract

The aim of this study was to investigate lifestyle habits in health and non-health science students from the University of Split, Croatia, and to evaluate their association with perceived stress and psychological well-being. We surveyed 783 students during February–March 2021. Hierarchical multiple regression analysis was used in the analysis. Health science students spent less time using screens, were sitting less, slept less, but also showed better compliance with the Mediterranean diet compared to non-health science students (10.6% vs. 5.9%). There were no differences in stress and well-being perception between the two study groups. Female gender, lack of sleep, less daily sitting time, anxiety, and lower optimism were associated with higher stress in non-health science students, while anxiousness and more sitting were found to be significant among health students. Well-being among health science students was positively associated with BMI, having had COVID-19, being refreshed after waking up during working days, Mediterranean diet adherence, health perception, optimism, and quality of life, and negatively with TV time and anxiousness. Shorter sleep duration on non-working days, feeling refreshed after waking up on non-working days, higher quality of life and optimism, and lower anxiousness were associated with higher well-being among non-health students. Identifying unhealthy lifestyle habits in students is essential for implementing targeted interventions to better their health outcomes.

## 1. Introduction

Students face various difficulties and strains that can lead these young people to develop unhealthy lifestyle habits [1,2]. The period of transition from high school to university education is a period of significant change and adjustment [3,4]. Several changes that may occur in lifestyle habits in students include shifts in eating habits, physical activity levels, sleep patterns, and stress perception, especially for those students who are living away from home for the first time, either in a rented apartment or a dormitory on campus. Previously published studies suggest that first-year students are particularly likely to modify their diets as they adapt to new surroundings and routines [2,5,6,7,8,9,10,11]. In addition, students can be faced with limited financial resources and the need to prepare their own meals [5,6,7,8,9,11,12]. During their study years, students tend to consume more fast food, snacks, sweetened drinks, and alcohol [6,7]. They also frequently skip meals, particularly breakfast [6,7,13]. However, unlike first-year students, older students have already gone through this initial adjustment period, so they are not exposed to these specific stressors relative to students at the beginning of their studies [14]. Previous research on eating behavior has identified several factors that may influence healthy food choices. These factors included taste preferences, lack of discipline, time constraints, improved nutrition knowledge and education, meal planning and involvement in meal preparation, social networks (such as social support from parents or colleagues), the physical environment (such as product prices, limited budgets, and availability of healthy and unhealthy foods), and macrosystems (such as media and advertising) [3]. 

Many students tend to overlook the risk of developing chronic diseases when making food choices, due to their young age. However, studies have shown that weight gain is a common occurrence during the study period, mostly due to unhealthy eating habits such as skipping meals, consuming inappropriate foods and snacks, and frequenting fast food outlets [12]. All these factors combined can increase the risk of poor health among students. Moreover, physical activity is also decreasing among students, which could be attributed to the increased time spent sitting during study and exam periods [12]. A lack of physical activity can negatively impact physical health, mood, and ability to cope with stress [15]. Physical inactivity is one of the challenges students face during the transition from high school to university education. In addition, students have pointed out obstacles to physical activity [16,17]. The most common obstacles were lack of time, busy schedule and other student obligations, inadequate exercise space, lack of motivation, season, and way of thinking [16,17]. 

Additionally, students often have irregular sleep schedules due to studying, exam deadlines, social activities and other obligations. Lack of quality sleep can result in fatigue, reduced concentration, weaker memory and general poor health [18,19]. Also, academic life can be demanding, causing some students to rely on caffeine or other stimulants to stay alert and concentrated. However, consuming too many stimulants can cause sleep issues, addiction, and overall negative health consequences [18]. 

The medical literature provides extensive evidence of how stress can directly and indirectly influence human health [20,21,22], as well as dietary habits [23,24]. According to the literature, stress is not a direct cause of health problems, but it is considered a risk factor that can trigger or influence the development, course, and outcomes of various diseases [20]. Numerous studies have identified stress as a leading risk factor for chronic non-communicable diseases, accounting for 75–90% of cases [20]. Stress can also indirectly affect health through risky behaviors and habits, such as smoking, excessive alcohol consumption, lack of physical activity, overeating, and sleep disturbances [25,26,27]. In a study conducted in 2019 at two university campuses in Korea, a significant difference was found in eating behaviors between students with high and low levels of perceived stress. Students who reported high levels of perceived stress were found to have increased unhealthy eating behaviors, such as consumption of ready-made meals [13]. On the other hand, results from a cohort study among German and Chinese students revealed that adopting healthy lifestyle habits could significantly improve overall well-being [28]. However, one of the challenges highlighted in this study is the need for long-term monitoring of individuals to accurately predict their mental health outcomes in the future [28].

COVID-19 brought long-term changes and serious disruptions in the social, psychological, and economic context [29,30,31]. Furthermore, changes titled the “new normal” globally influenced lifestyle habits and mental health [29,30,31,32,33]. COVID-19 also brought great changes in the education system. By decision of the state authorities, education that took place in schools and universities through personal “face to face” contact was switched to distance learning [31,34,35,36]. During this time, the organization and execution of lectures, seminars and practices was a great challenge for students, as well as for the teachers [35,36]. In addition, health science students attended most of their theoretical courses online, while the practical part of the courses was completed in health care facilities in accordance with epidemiological recommendations [36]. A quick adjustment among teachers and students was necessary to avoid even greater disruption to the education process [35]. However, the consequences of these changes in the educational system are evidenced by a published study from Spain that examined the prevalence of psychological symptoms and burnout among first-year students (health and non-health science students), and the results of this study showed that significant psychological distress was present among all students [37]. The quarantine period undoubtedly affected the quality of life, perceived stress, and feelings of happiness and optimism about the future in young people [38]. 

Health science students, as future health care workers, are expected to educate their patients on the importance of maintaining a healthy diet and engaging in regular physical activity, as well as aiming for restorative sleep. Therefore, it is crucial that these students are well-informed about the benefits of a balanced diet and exercise during their education. However, multiple studies have shown that health science students do not always put their knowledge into practice [16,17,39,40,41,42,43,44,45,46,47,48,49,50,51,52]. On the other hand, non-health science students may be less aware of and less focused on healthy eating, physical activity, as well as regular sleeping. Additionally, non-health science students may experience stress, but they may not have the same level of understanding regarding the significance and techniques of stress management [53]. 

Adolescence and young adulthood represent a significant transitional period that is challenging and critical for young people. Moreover, this period is of utmost importance for public health since lifestyle habits that develop during this period can have a lasting impact on an individual’s lifetime health outcomes [4,54,55]. Early identification of unhealthy habits is crucial for applying appropriate intervention measures, promoting population health, and preventing chronic diseases in the future. Given all these points, this study aimed to compare the lifestyle habits, perceived stress and psychological well-being among health and non-health science students from the University of Split, Croatia. Additionally, we aimed to identify students’ characteristics associated with perceived stress level and psychological well-being, while assessing the differences in these mental health indicators between health and non-health science students.

## 2. Materials and Methods

### 2.1. Study Design and Participants

This cross-sectional study was conducted in Split, Croatia, during the 2020/2021 academic year (February–March 2021). An invitation to participate in this study was sent to seven faculty institutions of the University of Split, but only four university institutions responded: University Department of Health Studies, Faculty of Science, Faculty of Chemistry and Technology, and Faculty of Humanities and Social Sciences. 

All students in Croatia have access to the Authentication and Authorization Infrastructure of the Science and Higher Education System (AAI@EduHR), and each student has an official email for communication with teachers. Therefore, teachers from four university institutions that agreed to participate sent their students a link to access the online survey for this study via the students’ official email addresses. No exclusion criteria were applied, while the main inclusion criterion was active student status. Data were collected anonymously using Google forms, which took on average 20 min to complete. Students were reminded three times at seven-day intervals in order to maximise the response rates. Students were informed of the purpose of the study and data collection before giving their responses. By completing the questionnaire, students gave their informed consent. 

In this study, 783 students participated out of a total of 3490 students who were contacted, resulting in a response rate of 22.4%. The sample size was calculated using the Raosoft online calculator [56]. Considering the student population of the University of Split at the time of data collection, which was around 20,000 students, with a confidence level of 95%, a margin of error of 5%, and a response distribution of 50%, the minimum appropriate sample size for this study was determined to be 377.

#### 2.1.1. Questionnaires

The questionnaire consisted of several sections as follows: (i) general demographic characteristics, (ii) lifestyle habits, (iii) adherence to the Mediterranean diet, (iv) perceived stress level, (v) health and self-perception assessment (general health, COVID-19, quality of life, happiness, anxiety and optimism), and (vi) psychological well-being.

The first part of the questionnaire included questions about age, gender, study information (institution, year of study, current education model (in person; online; hybrid model; or currently no classes, studying for the exam deadlines)), smoking habits (possible responses were: yes; ex-smoker; never smoked), alcohol consumption (possible responses were: no; occasionally; every day), and information on body weight, height, and time elapsed (in days) since the last body weight measurement. Body mass index (BMI) was calculated using the standard formula: BMI = weight (kg)/height (m^2^). Students were also asked about the average time they spend daily watching TV, using mobile phones (including calls and the Internet), time spent using the computer (for studying and entertainment), and their usual studying time per day.

##### Lifestyle Habits Assessment

The lifestyle habits questionnaire included: (i) sleeping habits, (ii) eating habits and (iii) physical activity.

Regarding sleeping habits, students were asked when they usually go to bed and when they wake up separately for working and non-working days, which was later used to calculate sleep duration. This part also included the way of waking up and the feeling of restfulness after awakening. Possible responses for the way of waking up in the morning were “alone” or “with an alarm clock”. Possible responses for feeling when waking up in the morning were “refreshed”, “partially tired and sleepy”, and “very tired and sleepy”. 

Eating habits were examined using questions about the number of meals per day (including the number of main meals and snacks, separately for weekdays and weekends), and snacking habits while watching TV or studying (possible responses were: yes, often; yes, sometimes; or no). Students were asked about cooking for themselves (possible responses were: yes, often; sometimes; no). We also asked students about their frequency of eating lunch in the student canteen/restaurant (possible responses were: every day; 3–4 times a week; 1–2 times a week; I do not eat in the student canteen).

Questions about physical activity included playing any sports and gym usage frequency (possible responses were: yes, several times a week; yes, once a week; rarely or never). Also, students were asked about the average time they spend daily sitting and walking (measured in minutes per day).

##### Mediterranean Diet Assessment

In order to examine adherence to the Mediterranean diet, this study used a validated and reliable Croatian version of the Mediterranean Diet Serving Score questionnaire (MDSS) [57]. MDSS is based on an updated model of the modern Mediterranean pyramid, which emphasizes food groups that should be consumed at each main meal (cereals, fruits, vegetables, olive oil), followed by food groups that should be consumed at least once or twice a day (dairy products and nuts), and food groups that should be consumed on a weekly basis (potatoes, legumes, eggs, fish, red meat) [58]. Briefly, the questionnaire includes 14 food groups, and individuals whose consumption is within the recommended range receive between 1 and 3 points for consumption of each food group per meal, day, or week. Individuals who do not reach the recommended intake receive 0 points, and there are no negative points [57]. The total score ranges from 0 to 24 points for adults and from 0 to 23 points for adolescents, since alcohol consumption is not considered appropriate in this age group [59]. According to the original study, people who reach a score of ≥13.5 are considered to be adherent to the Mediterranean diet [59]. In the Croatian version of the MDSS, participants who had 14 or more points were considered to be adherent to the Mediterranean diet [57]. For adults, 1 point was added for alcohol consumption (5–25 g/day for women or 25–50 g/day for men; or 2 decilitres of red wine for men and 1 decilitre for women). In the Croatian version of the MDSS we did not include beer in the group of fermented beverages, as originally proposed [59]. Instead, we included only the consumption of wine, which corresponds to the updated Mediterranean pyramid [58]. In addition, the question about consumption of sugar-sweetened juices and beverages was included as a separate question in the questionnaire, but was assessed within the group of sweets, as proposed in the original study [59].

##### Perceived Stress Level Assessment

The Perceived Stress Scale—10 (PSS-10) is a questionnaire consisting of 10 statements about the participants’ thoughts and feelings during the last month [60]. Quantitative assessment is based on a 5-point Likert scale with possible scores from 0 to 4 points for each of the questions (possible responses are: never; almost never; sometimes; often; very often). Adding up the scores of all 10 responses gives a total score that indicates the level of perceived stress in the last month. The maximum possible score for the questionnaire is 40, and a higher score indicates a higher stress level [60]. According to the original study, the PSS-10 is a short, reliable, and valid questionnaire with adequate internal consistency (Cronbach α = 0.85) [60]. In this study, the Croatian version of the PSS-10 was used, whose reliability and validity were previously confirmed, as well as its internal consistency (Cronbach α = 0.88) [61]. 

##### Health and Self-Perception Assessment

This section consisted of several questions about self-assessment of health, quality of life, feelings of anxiety, optimism, and happiness. A 10-point Likert scale was used for assessment. For the question assessing health, the possible range of responses was from 0 to 10 (0 = very ill, 10 = completely healthy). For the question assessing quality of life, the possible responses ranged from 0 = extremely low to 10 = excellent quality of life. Similarly, for feelings of anxiety, the possible responses ranged from 0 = not at all to 10 = extremely anxious/worried. Regarding feelings of happiness, the possible responses ranged from 0 = not at all to 10 = extremely happy, similar to the question about the assessment of optimism about the future (0 = not at all optimistic, 10 = extremely optimistic). 

Regarding health status, students were asked whether they have any chronic diseases (possible responses were: yes or no). An additional question was about having been diagnosed with COVID-19 (possible responses were: yes, no or I don’t know).

##### Psychological Well-Being

The Warwick–Edinburgh Mental Well-being Scale (WEMWBS) was used to examine students’ mental well-being. The WEMWBS consists of 14 positive statements that relate to how participants felt in the last two weeks [62]. It examines the major components of psychological functioning and well-being, including positive influences (feelings of optimism, cheerfulness, and relaxation) and positive functioning (energy, clarity of thought, self-confidence, autonomy, interest in other people or things) [62]. Possible responses to each of the statements are: never; rarely; sometimes; often; always, all the time. By summing the answers, the total score indicates a sense of well-being, and by applying the cut-off values, the score can be interpreted as low (≤40 points), medium (41 to 59 points), and high well-being (≥60 points) [63]. The total score of the questionnaire is 70, and the lowest score is 14 [62]. The reliability and validity of the WEMWBS questionnaire was assessed in populations of university students and adults, and members of national minorities, as well as in persons receiving mental health services and their caregivers [62]. The WEMWBS questionnaire proved to be a reliable and valid questionnaire with high values of internal consistency in the student population (Cronbach’s α = 0.89) and in the adult population (Cronbach’s α = 0.91) [62]. 

### 2.2. Statistical Analysis

Numerical data were represented using the median with interquartile range (IQR), while categorical data were presented using absolute frequency with percentage. Distribution of numerical variables was evaluated using the Kolmogorov–Smirnov test. As the numerical variables exhibited a non-normal distribution, a Mann–Whitney U test was employed for comparison between groups. The comparison between categorical variables was performed using chi-square and Fisher’s exact tests, as appropriate. Hierarchical multiple regression analysis was used to determine the association between students’ demographic characteristics (block 1 variables), lifestyle habits (block 2), and self-perception (block 3) with stress perception (PSS-10) and well-being (WEMWBS scores). For each of the two outcomes (stress and well-being) three models were created, one including all students, and two separate models for health science students and non-health science students, with the same set of predictor variables: gender, age, BMI, residence, living arrangements, and COVID-19 diagnosis (included in block 1), smoking, TV watching time, computer use, mobile phone use time, studying time, sleep duration, feeling refreshed after sleep, eating lunch at student canteen, cooking, MDSS score, daily sitting time, daily walking time, gym usage, recreation (included in block 2), and health perception, quality of life, anxiousness, optimism, well-being and stress perception (included in block 3).

The data were analysed using IBM SPSS Statistics for Windows, Version 20 (Armonk, NY, USA: IBM Corp.), and the results were interpreted with a significance level set at *p* < 0.05.

## 3. Results

The study involved a total of 783 students from the University of Split from four university institutions: the University Department of Health Studies (N = 292, 37.3%), Faculty of Science (N = 231, 29.5%), Faculty of Humanities and Social Sciences (N = 215, 27.5%), and Faculty of Chemistry and Technology (N = 45, 5.7%). For the purposes of this study, students were grouped into a health science group (University Department of Health Studies, N = 292, 37.3%) and a non-health science group of students (the other three faculties, N = 491, 62.7%). Median age was 21 (IQR 20–23), and the majority of the participants were female (84.8%) (Table 1).

Health science students were on average older (21, IQR 20–26 vs. 21, IQR 20–23), and a higher proportion of participants were involved in the first two study years. They predominantly had combined classes (67.8%), while non-health science students predominantly had no classes at all at the time of data collection (51.5%). All of these differences were statistically significant (all *p* < 0.001). On the other hand, there were no significant differences in gender composition, current residence, living arrangements or presence of chronic diseases between health science and non-health science students. At the time of data collection, 24% of health science students and 16.1% of non-health science students had been diagnosed with COVID-19 (Table 1).

**Table 1:** General characteristics of the participants in the overall sample and in the subgroups of health and non-health science students.

	Overall Sample (N = 783)	Non-Health Science Students (N = 491)	Health Science Students (N = 292)	*p*
Age (years); median (IQR)	21 (20–23)	21 (20–23)	21 (20–26)	<0.001 #
Gender; N (%)				
Females	664 (84.8)	414 (84.3)	250 (85.6)	0.681 †
Males	119 (15.2)	77 (15.7)	42 (14.4)	
Study year; N (%)				
1st	231 (29.5)	117 (23.8)	114 (39)	<0.001 ^§^
2nd	245 (31.3)	144 (29.3)	101 (34.6)	
3rd	168 (21.5)	107 (21.8)	61 (20.9)	
4th	78 (10)	77 (15.7)	1 (0.3)	
5th	58 (7.4)	43 (8.8)	15 (5.1)	
6th	3 (0.4)	3 (0.6)	0 (0)	
Current residence; N (%)				
City	540 (69)	350 (71.3)	190 (65.1)	0.165 †
Semi-urban	143 (18.3)	85 (17.3)	58 (19.9)	
Village	100 (12.8)	56 (11.4)	44 (15.1)	
Living with; N (%)				
Alone	90 (11.5)	56 (11.4)	34 (11.6)	0.112 †
In a family with one additional member	69 (8.8)	45 (9.2)	24 (8.2)	
In a family with ≥3 members	473 (60.4)	308 (62.7)	165 (56.5)	
With a partner	62 (7.9)	30 (6.1)	32 (11)	
At university campus	89 (11.4)	52 (10.6)	37 (12.7)	
Method of class attendance; N (%)				
In person	12 (1.5)	12 (2.4)	0 (0)	<0.001 ^§^
Online	144 (18.4)	50 (10.2)	94 (32.2)	
Combined (online and in person)	374 (47.8)	176 (35.8)	198 (67.8)	
Currently no classes, studying for exam deadlines	253 (32.3)	253 (51.5)	0 (0.0)	
Chronic disease; N (%)				
Yes	97 (12.4)	57 (11.6)	40 (13.7)	0.433 †
No	686 (87.6)	434 (88.4)	252 (86.3)	
Have you been diagnosed with COVID-19?; N (%)				0.001 †
Yes	149 (19.0)	79 (16.1)	70 (24.0)	
No	386 (49.3)	235 (47.9)	151 (51.7)	
I don’t know	248 (31.7)	177 (36.0)	71 (24.3)	

IQR—interquartile range; †—χ^2^-test; #—Mann–Whitney U test; ^§^—Fisher’s exact test.

Students were then compared according to different lifestyle habits (Table 2). There were no differences in smoking prevalence, alcohol intake or in BMI between study groups. Compared to health science students, non-health science students studied more (*p* < 0.001), and used computer/tablet (*p* < 0.001) and mobile phones (*p* = 0.023) for about 1 h longer. Regarding sleeping habits, non-health science students slept longer during working days (8, IQR 7.5–9 vs. 8, IQR 7–8.5, *p* < 0.001), and more often woke up without an alarm clock (*p* = 0.003), compared to health sciences students. However, fewer non-health science students felt refreshed after waking up on non-working days (43.2% vs. 56.8%; *p* < 0.001). Eating habits were also significantly different between groups. Health science students had on average higher MDSS scores (7, IQR 5–10 vs. 6, IQR 4–9, *p* < 0.001, Table 1), higher MD compliance (10.6% vs. 5.9%, *p* = 0.018), cooked for themselves more frequently (41.1% vs. 28.5%, *p* = 0.001), and the majority of them did not eat at the student canteen (70.5% vs. 50.1%, *p* < 0.001). In addition, health science students reported slightly higher compliance for consuming olive oil (20.8% vs. 11.6%), nuts (26.4% vs. 11.9%), fruits (20.9% vs. 15.3%), and fish (29.5% vs. 21.0%) (Figure S1). They also reported having more meals per day during weekends, but the difference was only visible when we analyzed differences between 5th and 95th quartiles (2–3 for health vs. 1–3 for non-health science students, *p* = 0.045). Non-health science students also reported lower physical activity. Compared to them, health science students used gyms more frequently (*p* = 0.022) and spent less time sitting during the day (*p* < 0.001) (Table 2). Other differences in lifestyle habits between study groups were not observed.

**Table 2:** Lifestyle habits in the overall sample and in subgroups of health and non-health science students.

	Overall Sample (N = 783)	Non-Health Science Students (N = 491)	Health Science Students (N = 292)	*p*
BMI (kg/m^2^); median (IQR)	21.7 (20.2–23.9)	21.6 (20.2–23.7)	21.9 (20.2–24.1)	0.112 #
Weighing (days ago); median (IQR)	15 (5–30)	15 (5–30)	15 (5–30)	0.516 #
Smoking; N (%)				
Yes	220 (28.1)	132 (26.9)	88 (30.1)	0.590 †
Ex-smokers	112 (14.3)	70 (14.3)	42 (14.4)	
Never smoked	451 (57.6)	289 (58.9)	162 (55.5)	
Alcohol N (%)				
No	216 (27.6)	133 (27.1)	83 (28.4)	0.722 †
Yes, occasionally	559 (71.4)	352 (71.7)	207 (70.9)	
Yes, every day	8 (1.0)	6 (1.2)	2 (0.7)	
Sedentary activity				
TV watching time (h/day); median (IQR)	1 (0–2)	1 (0–2)	1 (0.5–2)	0.122 #
Computer/tablet use time (h/day); median (IQR)	2.5 (1–4)	3 (1–5)	2 (1–4)	<0.001 #
Mobile phone use time (h/day); median (IQR)	3.5 (2–5)	4 (2.5–5)	3 (2–5)	0.023 #
Studying time (h/day); median (IQR)	2 (1.5–4)	3 (2–4)	2 (1–3)	<0.001 #
Sleeping habits during working days				
Sleep duration on working days (h); median (IQR)	8 (7–9)	8 (7.5–9)	8 (7.0–8.5)	<0.001#
Waking up on working days; N (%)				
Alone	137 (17.5)	101 (20.6)	36 (12.3)	0.003 †
Alarm clock	646 (82.5)	390 (79.4)	256 (87.7)	
Feeling after waking up on working days; N (%)				
refreshed	95 (12.1)	60 (12.2)	35 (12.0)	0.432 †
somewhat tired and sleepy	522 (66.7)	320 (65.2)	202 (69.2)	
extremely tired and sleepy	166 (21.2)	111 (22.6)	55 (18.8)	
Sleeping habits during non-working days				
Sleep duration on non-working days (h); median (IQR)	9 (8.0–9.5)	9 (8.0–9.5)	9 (8.0–9.5)	0.225 #
Waking up on non-working days; N (%)				
Alone	623 (79.6)	387 (78.8)	236 (80.8)	0.522 †
Alarm clock	160 (20.4)	104 (21.2)	56 (19.2)	
Feeling after waking up on non-working days; N (%)				
refreshed	378 (48.3)	212 (43.2)	166 (56.8)	<0.001 †
somewhat tired and sleepy	345 (44.1)	231 (47.0)	114 (39.0)	
extremely tired and sleepy	60 (7.7)	48 (9.8)	12 (4.1)	
Eating habits				
MDSS score (points); median (IQR)	7 (5–9)	6 (4–9)	7 (5–10)	<0.001 #
Mediterranean diet compliance (MDSS ≥ 14); N (%)				
No	723 (92.3)	462 (94.1)	261 (89.4)	0.018 †
Yes	60 (7.7)	29 (5.9)	31 (10.6)	
Meals per day (during working days); median (IQR)	3 (2–3)	3 (2–3)	3 (2–3)	0.645 #
Snacking between meals (during working days); median (IQR)	2 (1–2)	2 (1–2)	1 (1–2)	0.338 #
Meals per day (during weekends); median (IQR)	3 (2–3)	3 (2–3)	3 (2–3)	0.045 #
Snacking between meals (during weekends); median (IQR)	2 (1–3)	2 (1–3)	2 (1–3)	0.717 #
Cooking; N (%)				
Yes, frequently	260 (33.2)	140 (28.5)	120 (41.1)	0.001 †
Sometimes	372 (47.5)	253 (51.5)	119 (40.8)	
No	151 (19.3)	98 (20)	53 (18.2)	
Lunch at the student canteen; N (%)				
Every day	110 (14.0)	81 (16.5)	29 (9.9)	<0.001 †
1–2 times a week	155 (19.8)	117 (23.8)	38 (13.0)	
3–4 times a week	66 (8.4)	47 (9.6)	19 (6.5)	
I don’t eat in the student canteen	452 (57.7)	246 (50.1)	206 (70.5)	
Physical activity				
Sports; N (%)				
Yes, several times a week	230 (29.4)	140 (28.5)	90 (30.8)	0.355 †
Yes, once a week	105 (13.4)	61 (12.4)	44 (15.1)	
Rarely or never	448 (57.2)	290 (59.1)	158 (54.1)	
Gym; N (%)				
Yes, several times a week	129 (16.5)	67 (13.6)	62 (21.2)	0.022 †
Yes, once a week	14 (1.8)	9 (1.8)	5 (1.7)	
Rarely or never	640 (81.7)	415 (84.5)	225 (77.1)	
Another type of recreation, such as dancing, yoga, pilates; N (%)				
Yes, several times a week	145 (18.5)	97 (19.8)	48 (16.4)	0.445 †
Yes, once a week	101 (12.9)	60 (12.2)	41 (14.0)	
Rarely or never	537 (68.6)	334 (68.0)	203 (69.6)	
Daily sitting (h/day); median (IQR) ^§^	5 (3–7)	5 (3.5–8)	3.5 (2.0–5.5)	<0.001 #
Daily walking (h/day); median (IQR) ^¥^	1.5 (1–2)	1.5 (1–2)	1.5 (1–2)	0.521 #

BMI—body mass index; IQR—interquartile range; †—χ^2^-test; #—Mann–Whitney U test; ^§^—N = 507 (of which 39.8% health science students); ^¥^—N = 686 (of which 36.4% health science students).

In analysis of self-perception, health science students reported better overall health perception (*p* = 0.001), higher quality of life (*p* = 0.001), higher happiness (*p* < 0.001) and optimism about the future (*p* < 0.001), and lower anxiousness (*p* < 0.001) (Table 3). Furthermore, they showed lower perceived stress (PSS-10 score: 21, IQR 18–23, vs. 22, IQR 19–25, *p* < 0.001), and higher psychological well-being (WEMWBS score: 52, IQR 43.5–57, vs. 47, IQR 39–54, *p* < 0.001) (Table 3).

**Table 3:** Self-perceived assessment, perceived stress, and psychological well-being in the overall sample and in subgroups of health and non-health science students.

	Overall Sample (N = 783)	Non-Health Science Students (N = 491)	Health Science Students (N = 292)	*p*
Self-perception assessment				
Self-rated health perception; median (IQR)	9 (8–10)	9 (7–9)	9 (8–10)	0.001 #
Quality of life; median (IQR)	7 (6–8)	7 (6–8)	8 (7–9)	0.001 #
Happiness; median (IQR)	7 (5–8)	7 (5–8)	7 (6–9)	<0.001 #
Anxiousness; median (IQR)	5 (3–8)	6 (3–8)	4 (2–7)	<0.001 #
Optimistic about future; median (IQR)	7 (5–8)	7 (5–8)	8 (6–9)	<0.001 #
Perceived stress				
Perceived stress score (PSS-10); median (IQR)	21 (19–25)	22 (19–25)	21 (18–23)	<0.001 #
Perceived stress category (PSS-10); N (%)				
low	29 (3.7)	18 (3.7)	11 (3.8)	<0.001 †
moderate	636 (81.2)	377 (76.8)	259 (88.7)	
high	118 (15.1)	96 (19.6)	22 (7.5)	
Psychological well-being				
Psychological well-being score (WEMWBS); median (IQR)	48 (41–55)	47 (39–54)	52 (43.5–57)	<0.001 #
Psychological well-being (WEMWBS); N (%)				
low	183 (23.4)	141 (28.7)	42 (14.4)	<0.001 †
moderate	512 (65.4)	313 (36.7)	199 (68.2)	
high	88 (11.2)	37 (7.5)	51 (17.5)	

IQR—interquartile range; WEMWBS—The Warwick–Edinburgh Mental Well-being Scale; †—χ^2^-test; #—Mann–Whitney U test.

Subsequently, a hierarchical multiple regression analysis was conducted to examine the association between lifestyle habits and stress perception (Table 4) and psychological well-being (Table 5), while controlling for confounding factors. In the overall sample, there were no differences between health science and non-health science students in stress perception (*p* = 0.626), while women were more likely to report higher stress perception (*p* = 0.001), the same as students who reported longer sleeping times during non-working days (*p* = 0.010), higher anxiousness (*p* < 0.001), and lower optimism about the future (*p* < 0.009; Table 4). 

There were several significant associations between students’ characteristics and habits and higher stress perception (higher PSS-10 score) among non-health science students. These included female gender (*p* < 0.001), longer sleep duration on non-working days (*p* = 0.045), not cooking (*p* = 0.010), shorter daily sitting time (*p* = 0.039), higher self-perceived anxiousness (*p* < 0.001), and lower optimism about the future (*p* = 0.014). Among health science students, more daily sitting time (*p* = 0.045) and higher self-perceived anxiousness (*p* < 0.001) emerged as significant predictors of higher PSS-10 scores (Table 4). 

**Table 4:** Association between lifestyle habits and perceived stress in the overall sample and in both study subgroups (hierarchical multiple regression analysis).

	All Students				Non-Health Science Students				Health Science Students			
	Beta	Beta 95% CI Lower Bound	Beta 95% CI Upper Bound	*p*	Beta	Beta 95% CI Lower Bound	95% Beta CI Upper Bound	*p*	Beta	Beta 95% CI Lower Bound	Beta 95% CI Upper Bound	*p*
Health science students (non-health science students were referent group)	−0.147	−0.740	0.445	0.626	-	-	-	-	-	-	-	-
Women (men were referent group)	1.248	0.495	2.001	0.001	1.686	0.694	2.678	0.001	0.561	−0.671	1.794	0.370
Age (years)	−0.014	−0.068	0.039	0.601	−0.079	−0.208	0.049	0.226	−0.003	−0.067	0.061	0.932
BMI (kg/m^2^)	0.031	−0.055	0.116	0.482	0.012	−0.101	0.125	0.834	0.105	−0.041	0.250	0.157
Semi-urban and village residence (city was referent)	−0.039	−0.588	0.509	0.888	−0.053	−0.790	0.684	0.887	−0.008	−0.869	0.854	0.986
Living alone (students living with family were referent group)	0.541	−0.270	1.352	0.191	0.632	−0.458	1.723	0.255	0.209	−1.066	1.484	0.747
Living at university campus (students living with family were referent group)	0.579	−0.374	1.531	0.233	0.376	−0.857	1.609	0.550	0.256	−1.461	1.972	0.769
Diagnosed with COVID-19 (not diagnosed and students not sure were referent)	0.027	−0.615	0.668	0.935	0.561	−0.327	1.449	0.215	−0.497	−1.468	0.474	0.315
Smoking (non-smokers and ex-smokers were referent group)	0.400	−0.162	0.963	0.163	0.338	−0.419	1.095	0.381	0.458	−0.442	1.357	0.318
TV watching time (h/day)	−0.142	−0.380	0.095	0.240	−0.038	−0.364	0.287	0.817	−0.236	−0.603	0.132	0.207
Computer/tablet use time (h/day)	−0.027	−0.139	0.085	0.634	0.004	−0.129	0.137	0.954	−0.045	−0.288	0.198	0.714
Mobile phone use time (h/day)	−0.056	−0.147	0.035	0.228	−0.067	−0.193	0.059	0.295	−0.067	−0.206	0.072	0.342
Studying time (h/day)	0.054	−0.074	0.182	0.410	0.023	−0.127	0.173	0.762	0.118	−0.185	0.421	0.444
Sleep duration on working days (h)	−0.115	−0.328	0.098	0.291	−0.058	−0.337	0.221	0.681	−0.123	−0.482	0.237	0.502
Sleep duration on non-working days (h)	0.308	0.072	0.543	0.010	0.333	0.007	0.658	0.045	0.229	−0.124	0.582	0.203
Feeling refreshed after waking up on working days (not refreshed was referent group)	−0.515	−1.341	0.311	0.221	−0.017	−1.091	1.057	0.975	−1.375	−2.747	−0.002	0.050
Feeling refreshed after waking up on non-working days	−0.260	−0.828	0.308	0.368	−0.251	−0.95	0.493	0.507	−0.474	−1.392	0.445	0.311
Lunch at the student canteen (days/week)	0.194	−0.073	0.461	0.154	0.233	−0.087	0.553	0.154	0.102	−0.419	0.624	0.700
Cooking sometimes (cooking frequently was referent group)	0.039	−0.569	0.648	0.899	0.437	−0.363	1.237	0.284	−0.304	−1.298	0.690	0.547
Cooking never (cooking frequently was referent group)	0.697	−0.120	1.513	0.094	1.430	0.334	2.516	0.010	0.014	−1.305	1.333	0.983
Mediterranean Diet Serving Score (MDSS)	0.054	−0.016	0.124	0.129	0.040	−0.055	0.134	0.409	0.050	−0.058	0.158	0.364
Daily sitting (h/day)	−0.040	−0.150	0.071	0.485	−0.149	−0.290	−0.007	0.039	0.198	0.004	0.392	0.045
Daily walking (h/day)	0.131	−0.100	0.362	0.266	0.087	−0.325	0.500	0.677	0.145	−0.140	0.430	0.318
Gym (never or rarely was referent group)	0.074	−0.593	0.741	0.828	−0.119	−1.048	0.809	0.801	0.088	−0.911	1.086	0.863
Another type of recreation (never or rarely was referent group)	−0.217	−0.761	0.326	0.432	−0.184	−0.900	0.533	0.614	−0.182	−1.071	0.708	0.688
Self-rated health perception	−0.067	−0.229	0.094	0.411	−0.164	−0.357	0.029	0.095	0.102	−0.230	0.433	0.547
Quality of life	−0.127	−0.319	0.065	0.194	−0.190	−0.433	0.053	0.125	−0.041	−0.371	0.289	0.805
Anxiousness	0.759	0.642	0.876	<0.001	0.824	0.669	0.978	<0.001	0.632	0.445	0.819	<0.001
Optimistic about future	−0.183	−0.320	−0.046	0.009	−0.209	−0.374	−0.043	0.014	−0.060	−0.333	0.212	0.664
Psychological well-being score (WEMWBS)	−0.013	−0.052	0.026	0.519	−0.001	−0.050	0.048	0.961	−0.053	−0.126	0.019	0.150
Full model summary	Durbin–Watson test = 1.965; R^2^ adjusted = 0.415				Durbin–Watson test = 1.915; R^2^ adjusted = 0.432				Durbin–Watson test = 2.027; R^2^ adjusted = 0.355			

Regarding WEMWBS scores in the overall sample, we recorded no difference between non-health science and health science students in well-being perception (*p* = 0.351; Table 5). Significant positive associations in the overall sample of students were found between well-being and feeling refreshed after waking up on both working days (*p* = 0.045) and non-working days (*p* = 0.003), quality of life (*p* < 0.001), and optimism (*p* < 0.001), while lower well-being perception was reported in students who were sleeping longer on non-working days (*p* = 0.004) and who were more anxious (*p* < 0.001).

Several factors were significantly associated with higher scores for well-being among non-health occupation students. These factors included shorter sleep duration on non-working days (*p* = 0.045), feeling refreshed after waking up on non-working days (*p* = 0.003), higher self-perceived quality of life (*p <* 0.001), and optimism about the future (*p* < 0.001), as well as lower self-perceived anxiousness (*p* < 0.001). In contrast, among health science students, higher BMI (*p* = 0.047), having been previously diagnosed with COVID-19 (*p* = 0.003), shorter TV-watching time (*p* = 0.031), feeling refreshed after waking up on working days (*p* = 0.030), higher Mediterranean Diet Serving Score (*p* = 0.010),higher self-rated health perception (*p* = 0.001), higher self-rated optimism about the future (*p* < 0.001), higher quality of life (*p <* 0.001), and lower self-perceived anxiousness (*p* < 0.001) were significantly associated with higher WEMWBS scores (Table 5).

**Table 5:** Association between lifestyle habits and psychological well-being in the overall sample and in both study subgroups (multivariate linear regression).

	All Students				Non-Health Students				Health Students			
	Beta	Beta 95% CI Lower Bound	Beta 95% CI Upper Bound	*p*	Beta	Beta 95% CI Lower Bound	95% Beta CI Upper Bound	*p*	Beta	Beta 95% CI Lower Bound	Beta 95% CI Upper Bound	*p*
Health science students (non-health science students were referent group)	0.511	−0.565	1.587	0.351	-	-	-	-	-	-	-	-
Women (men were referent group)	0.346	−1.032	1.724	0.622	0.493	−1.380	2.366	0.605	0.664	−1.392	2.720	0.525
Age (years)	−0.003	−0.101	0.095	0.954	0.043	−0.197	0.284	0.724	−0.040	−0.146	0.066	0.460
BMI (kg/m^2^)	−0.053	−0.209	0.103	0.506	−0.154	−0.364	0.056	0.150	0.245	0.003	0.486	0.047
Semi-urban and village residence (city was referent)	0.318	−0.678	1.314	0.531	0.076	−1.299	1.451	0.914	0.789	−0.643	2.222	0.279
Living alone (students living with family is referent group)	−0.506	−1.981	0.968	0.500	−1.272	−3.306	0.762	0.220	0.812	−1.311	2.936	0.452
Living at university campus (students living with family is referent group)	−0.685	−2.416	1.046	0.437	−0.190	−2.492	2.111	0.871	−2.438	−5.284	0.409	0.093
Diagnosed with COVID-19 (not diagnosed and students not sure were referent)	1.068	−0.095	2.23	0.072	0.140	−1.520	1.799	0.869	2.454	0.860	4.048	0.003
Smoking (non-smokers and ex-smokers are referent group)	0.328	−0.695	1.351	0.529	0.156	−1.258	1.570	0.828	0.165	−1.338	1.667	0.829
TV watching time (h/day)	−0.280	−0.712	0.151	0.202	−0.042	−0.650	0.565	0.891	−0.670	−1.279	−0.062	0.031
Computer/tablet use time (h/day)	−0.119	−0.322	0.084	0.250	−0.034	−0.281	0.214	0.790	−0.262	−0.666	0.142	0.203
Mobile phone use time (h/day)	0.001	−0.166	0.165	0.999	0.148	−0.087	0.383	0.216	−0.220	−0.451	0.010	0.061
Studying time (h/day)	0.024	−0.209	0.257	0.838	−0.051	−0.332	0.229	0.718	0.298	−0.206	0.802	0.246
Sleep duration on working days (h)	−0.073	−0.461	0.315	0.711	−0.108	−0.628	0.413	0.684	−0.289	−0.888	0.309	0.342
Sleep duration on non-working days (h)	−0.623	−1.050	−0.196	0.004	−0.622	−1.230	−0.014	0.045	−0.266	−0.856	0.323	0.374
Feeling refreshed after waking up on working days (not refreshed is referent group)	1.534	0.035	3.032	0.045	0.760	−1.243	2.763	0.456	2.534	0.250	4.819	0.030
Feeling refreshed after waking up on non-working days	1.571	0.545	2.597	0.003	2.097	0.722	3.472	0.003	0.310	−1.223	1.844	0.691
Lunch at the student canteen (days/week)	−0.286	−0.770	0.199	0.248	−0.386	−0.983	0.212	0.205	−0.356	−1.224	0.512	0.420
Cooking sometimes (cooking frequently is referent group)	−0.914	−2.018	0.189	0.104	−1.062	−2.553	0.430	0.162	−1.270	−2.920	0.380	0.131
Cooking never (cooking frequently is referent group)	−0.614	−2.099	0.871	0.417	−0.827	−2.868	1.213	0.426	−0.474	−2.672	1.724	0.672
Mediterranean Diet Serving Score (MDSS)	0.102	−0.025	0.229	0.115	0.063	−0.113	0.240	0.481	0.235	0.057	0.414	0.010
Daily sitting (h/day)	−0.101	−0.303	0.101	0.326	−0.198	−0.463	0.066	0.141	0.111	−0.215	0.436	0.503
Daily walking (h/day)	0.058	−0.361	0.478	0.785	0.445	−0.323	1.213	0.256	−0.204	0.680	1.272	0.400
Gym (never or rarely is referent group)	−0.288	−1.499	0.924	0.641	0.408	−1.325	2.141	0.644	−1.428	−3.084	0.227	0.091
Another type of recreation (never or rarely is referent group)	0.035	−0.853	1.023	0.944	−0.347	−1.684	0.991	0.611	1.109	−0.368	2.586	0.141
Self-rated health perception	0.087	−0.206	0.380	0.559	−0.109	−0.470	0.251	0.551	0.901	0.359	1.443	0.001
Quality of life	1.515	1.183	1.847	<0.001	1.663	1.234	2.091	<0.001	1.094	0.560	1.628	<0.001
Anxiousness	−1.261	−1.478	−1.044	<0.001	−1.335	−1.631	−1.039	<0.001	−1.021	−1.334	−0.708	<0.001
Optimistic about future	1.073	0.834	1.311	<0.001	0.994	0.697	1.292	<0.001	1.203	0.773	1.633	<0.001
Perceived stress score (PSS)	−0.043	−0.173	0.087	0.519	−0.004	−0.175	0.167	0.961	−0.148	−0.350	0.054	0.150
Full model summary	Durbin-Watson test = 1.859; R^2^ adjusted = 0.635				Durbin-Watson test = 1.900; R^2^ adjusted = 0.610				Durbin-Watson test = 1.733; R^2^ adjusted= 0.655			

## 4. Discussion

This study revealed that health and non-health science students had comparable lifestyle habits, with a couple of exemptions, such as the amount of time spent using screens and in sedentary activity, and Mediterranean diet adherence. However, there were no differences in perceived stress level or in psychological well-being between health and non-health science students in the regression analysis. 

Furthermore, certain lifestyle habits and demographic characteristics were found to be associated with the students’ perceived stress and psychological well-being. These included female gender, length of sleep during non-working days, and feelings of anxiety and optimism about the future, which were found to be associated with perceived stress in the overall sample of students. Similarly, several lifestyle habits were also associated with psychological well-being in all students, including sleep duration on non-working days, feeling refreshed after waking up during working and non-working days, quality of life, anxiety, and optimism about the future. 

Among other interesting results, we found that female students in the non-health science subgroup were more likely to experience higher level of perceived stress, while health science students did not show this pattern. We assume that this result stems from differences in ways of coping with stress between men and women [64,65]. Previous studies also indicated that female students tend to experience higher levels of stress compared to male students [64,65,66,67,68]. A study which aimed to determine the prevalence and predictors of mental health among students conducted in Poland, Slovenia, the Czech Republic, Ukraine, Russia, Germany, Turkey, Israel, and Colombia also found that female gender was a significant predictor of PSS-10 score [69]. In addition, that study revealed high levels of perceived stress and mild symptoms of anxiety and depression in students from all nine countries [69]. Our study found that the majority of students reported a moderate level of perceived stress, which was similar to the findings of a multicentric study conducted in seven countries [70]. However, one in five non-health science students reported high levels of stress, compared to 7.5% of health science students. This could be explained by the ongoing exam period for non-health science students only during data collection. However, this difference between groups was not confirmed in linear regression. On the other hand, health science students reported a higher level of perceived anxiety compared to non-health science students. 

There is a plethora of evidence in medical literature that shows how stress can adversely impact human health, both directly and indirectly [20,22]. Health science students often encounter stressful situations during clinical exercises, which can include dealing with various health conditions, diseases, and even deaths of patients. These added stressors can compound the academic stress they already face. Also, according to the available literature, there is a difference in relationships with patients and stressful situations between different fields of health science studies, such as medicine, nursing, midwifery and physiotherapy [71,72]. Likewise, health science students often report higher levels of stress compared to non-health science students [66,67,73,74,75]. However, the results of our study did not concur with these previous findings. 

Regarding sleeping habits, there was an association between duration of sleep on non-working days and perceived stress and well-being. We found a significant association between duration of sleep and well-being in non-health science students. Based on our findings, non-health science students tend to sleep slightly longer and wake up more often on their own during weekdays rather than relying on an alarm clock, unlike health science students. However, we did not assess sleep quality in this study. Instead, we asked students about their feeling of refreshment after a night’s sleep. Our results revealed no significant differences between student groups in the percentage of students who reported feeling rested and refreshed during working days, which was quite low (12%), but more non-health science students reported being extremely tired and sleepy during non-working days (9.8%), compared to health science students (4.1%). 

According to the guidelines of the National Sleep Foundation, the appropriate duration of sleep for young people and adults is 7–9 h [76]. However, despite this recommendation, it is common for students to have irregular sleep patterns. This often involves sleeping longer during non-working days to at least partially compensate for the lack of sleep they experienced during working days [77,78,79,80]. One study aimed to determine the differences between medical and social science students in perceived stress levels, subjective quality of life and prosocial attitude. Major differences were not observed, but medical students were found to be more fatigued and had a greater sense of self-worth compared to social science students [81]. 

Although daily sitting was not found to be a significant predictor of stress in the overall study group, it was observed that non-health science students spent more time sitting (median: 5, IQR: 3.5–8) when compared to health science students (median: 3.5, IQR: 2.0–5.5). This difference is most likely due to the different schedules of classes during the period of data collection. This could have also affected another interesting finding regarding a difference in the type of association between sitting time and stress perception in the two study groups, in that we identified a negative association in non-health students, while a positive association was found in health science students. 

Being physically active is challenging for students transitioning from high school to higher education. Previous studies suggest that students often face obstacles to physical activity. Unfortunately, inactivity increases the risk of death by 20% to 30% compared to people who are sufficiently physically active [82,83]. Inactive people should devote at least three days a week to intense aerobic physical activity, and equally to physical activity for strengthening muscles and bones. The World Health Organization recommends adults aged 18 to 64 have at least 150 min of moderate-intensity exercise or 75 min of intensive exercise per week [82]. However, a study from the UK that involved medical students revealed a lack of knowledge about guidelines related to physical activity [47]. This raised the level of awareness and led to the conclusion that it is necessary to improve the education of medical students about the importance of physical activity [47]. Contrary to the previous study, among nursing students from Ireland, 60.2% of students were familiar with the Irish guidelines for physical activity, while 94.6% reported that they were aware that the guidelines differed for children, adults, and elderly people [84]. The majority of students (83.7%) correctly stated the physical activity guidelines for Irish adults. The survey revealed that most nursing students (94.7%) had learned about the benefits of physical activity for health. However, only 45.7% of students believed they were qualified enough to counsel the general population, while only 31.9% believed they were capable of counselling patients suffering from chronic non-communicable diseases [84]. Fitness programs within universities should be offered and adjusted over time, so that students can find enough time for regular physical activity.

Our study found some differences in eating habits among the student groups. Even though adherence to the Mediterranean diet was very low, health science students had greater adherence to the Mediterranean diet, they cooked more often, and the vast majority of them did not eat in the student canteen when compared to non-health science students. Previous studies found that adherence to the Mediterranean diet can improve sleep quality. Researchers have noted that consuming fruits, vegetables, grains, and legumes has a positive impact on sleep [77,78]. On the other hand, previous studies have shown that a lack of sleep contributes to increased concentrations of the appetite-stimulating peptide ghrelin [78,85,86]. Therefore, studies have shown that people who experience poor sleep tend to consume high-calorie diets more frequently, and have lower adherence to the Mediterranean diet [77,78,85,87]. 

Also, our study showed an association between psychological well-being and adherence to the Mediterranean diet in health science students. This correlates with the findings of Lo Moro and colleagues, who discovered a statistically significant relationship between adherence to the Mediterranean diet and total score on the WEMWBS questionnaire among students from the University of Turin in Italy [63]. They showed moderate well-being among students, with an average value of 46 points (IQR 41–46) [63]. This result is somewhat lower compared to our results, since health science students had 52 points as their average result for mental well-being, while non-health science students scored 47 points. A study conducted by Zollars and colleagues showed that regular mindfulness meditation practice can significantly enhance mental health, alleviate stress, and promote well-being in individuals pursuing rigorous health science programs [88]. Many studies deal with these problems in health sciences students, especially with the problem of anxiety and depression among medical students, which requires greater attention due to its significant implications, and because approximately one in three medical students in the world suffers from anxiety [89] and depression [90], a prevalence rate that is much higher than in the general population. Some study programs introduced mindfulness training for health science students [91], which suggested that mindfulness may serve to regulate low positive emotionality, poor mood regulation, and negative self-concept [92].

Adolescence and young adulthood as a transitional period of growing up represent a great challenge for young people, and are also important from the perspective of public health, considering that during the adolescent period different lifestyle habits develop, and some of them remain for the rest of the lifespan [54,55]. It is important to identify unhealthy lifestyle habits as early as possible in order to take appropriate intervention measures and thereby promote the health of the population and prevent chronic diseases in the future. Unfortunately, the COVID-19 epidemic exacerbated unhealthy behaviours that were already prevalent among young people, and may negatively impact their mental health [33,38,93,94,95,96,97]. Understanding the impact of life circumstances on students’ lifestyle habits can help us develop strategies to promote their mental health. It is also important to implement interventions that focus on maintaining a healthy lifestyle to preserve health and manage stress. This includes various factors that contribute to overall well-being. Considering the fact that this study was conducted after the easing of epidemiological measures in Croatia, it would certainly be necessary to repeat the study in order to compare the obtained results with the current situation.

Our study suffers from some limitations. Firstly, this is a cross-sectional study, making it impossible to establish a causal relationship. Secondly, the data were collected using an online questionnaire because of the epidemic caused by the SARS-CoV-2 virus, which made it impossible to collect data in any other way. In addition, women predominate in our sample. As one of the possible reasons, men tend to have a lower propensity to participate in online research compared to women. According to a study by Park K. et al., online survey participation rates tend to increase when the topic is attractive to the participants [98]. We believe that the low response rate in our survey (22.4%) was also influenced by the large number of requests for online questionnaire research related to COVID-19 that were carried out during that period. To address this issue, we sent reminders to encourage greater participation. 

Numerous previous studies have explored the factors associated with stress and well-being among students, as documented in the literature [4,99]. Only a few studies compared health and non-health science students and hence provided a comprehensive understanding of their differences [53,100,101]. Consequently, this study’s value lies in identifying the distinct factors that are associated with perceived stress and well-being in each group separately. 

## 5. Conclusions

Protecting students’ health is a big challenge, and it is crucial to ensure their well-being and academic success during study. Educating students about healthy lifestyle habits through lectures, workshops or informative materials can help them become more aware of the importance of caring for their health. Additionally, establishing a student health center and psychological counselling center on campus could help students deal with stress, anxiety, depression, and other mental challenges during this distinct and demanding phase of life. 

Moreover, supporting students who want to quit smoking or reduce alcohol or drug use, and encouraging healthy lifestyles, such as exercise, good sleep hygiene, and a balanced diet, would contribute to the overall health of students. A combination of these strategies could help create a campus environment that encourages and supports both students’ physical health and their well-being. It is crucial to continuously monitor the needs of students and adjust measures to ensure that their health and well-being needs are met.

This is one of the rare studies that compared lifestyle habits between health and non-health science students. It is important to comprehend how life circumstances affect the lifestyle of students, particularly those studying in the field of health sciences, to develop strategies that can promote their overall and mental health. This highlights the necessity for interventions to sustain healthy lifestyle habits that preserve good health and manage stress. Additionally, it is essential to address the factors that have led to negative changes in eating habits. 

## Data Availability

All relevant data are contained within the article. The data presented in this study are available upon reasonable request from the corresponding author.

## Supplementary Materials

The following supporting information can be downloaded at: https://www.mdpi.com/article/10.3390/nu16050620/s1, Figure S1: Mediterranean diet compliance between health and non-health science students.
