# Supplementary figures and images for: Differences between Health and Non-Health Science Students in Lifestyle Habits, Perceived Stress and Psychological Well-Being: A Cross-Sectional Study
Source: Nutrients. 2024 Feb 23;16(5):620. doi: 10.3390/nu16050620 (PMC10935260; doi:10.3390/nu16050620)

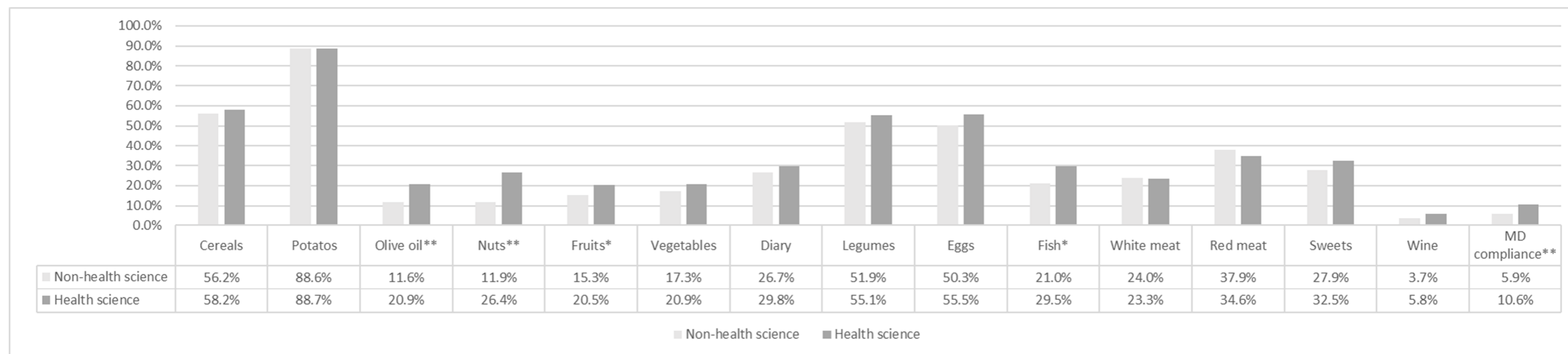

\*  $p < 0.05$ ; \*\*  $p < 0.001$

**Figure S1.** Mediterranean diet compliance between Health and Non-health science students.

Supplement: Supplementary file 1 [file nutrients-16-00620-s001.zip › nutrients-2846616-supplementary.pdf]
